# Immunogenicity of COVID-19 booster vaccination in IEI patients and their one year clinical follow-up after start of the COVID-19 vaccination program

**DOI:** 10.3389/fimmu.2024.1390022

**Published:** 2024-04-18

**Authors:** Leanne P. M. van Leeuwen, Marloes Grobben, Corine H. GeurtsvanKessel, Pauline M. Ellerbroek, Godelieve J. de Bree, Judith Potjewijd, Abraham Rutgers, Hetty Jolink, Frank L. van de Veerdonk, Marit J. van Gils, Rory D. de Vries, Virgil A. S. H. Dalm, VACOPID Research Group, Eric C.M. van Gorp

**Affiliations:** ^1^ Department of Viroscience, Erasmus MC University Medical Center Rotterdam, Rotterdam, Netherlands; ^2^ Travel Clinic, Erasmus MC University Medical Center Rotterdam, Rotterdam, Netherlands; ^3^ Department of Medical Microbiology and Infection Prevention, Amsterdam Institute for Infection and Immunity, Amsterdam UMC, University of Amsterdam, Amsterdam, Netherlands; ^4^ Department of Internal Medicine, Infectious Diseases, University Medical Center Utrecht, Utrecht, Netherlands; ^5^ Department of Infectious Diseases, Amsterdam UMC, Amsterdam, Netherlands; ^6^ Department of Internal Medicine, Division Clinical Immunology, Maastricht UMC, Maastricht, Netherlands; ^7^ Department of Rheumatology and Clinical Immunology, UMC Groningen, Groningen, Netherlands; ^8^ Department of Infectious Diseases, Leiden University Medical Center, Leiden, Netherlands; ^9^ Department of Internal Medicine, Radboud University Medical Center Nijmegen, Nijmegen, Netherlands; ^10^ Department of Internal Medicine, Division of Allergy & Clinical Immunology, Erasmus MC University Medical Center Rotterdam, Rotterdam, Netherlands; ^11^ Department of Immunology, Erasmus MC University Medical Center Rotterdam, Rotterdam, Netherlands

**Keywords:** inborn errors of immunity, primary immunodeficiency disorders, SARS-CoV-2, mRNA-1273 COVID-19 vaccine, booster vaccination, immunogenicity, antibody response, T-cell response

## Abstract

**Purpose:**

Previous studies have demonstrated that the majority of patients with an inborn error of immunity (IEI) develop a spike (S)-specific IgG antibody and T-cell response after two doses of the mRNA-1273 COVID-19 vaccine, but little is known about the response to a booster vaccination. We studied the immune responses 8 weeks after booster vaccination with mRNA-based COVID-19 vaccines in 171 IEI patients. Moreover, we evaluated the clinical outcomes in these patients one year after the start of the Dutch COVID-19 vaccination campaign.

**Methods:**

This study was embedded in a large prospective multicenter study investigating the immunogenicity of COVID-19 mRNA-based vaccines in IEI (VACOPID study). Blood samples were taken from 244 participants 8 weeks after booster vaccination. These participants included 171 IEI patients (X-linked agammaglobulinemia (XLA;N=11), combined immunodeficiency (CID;N=4), common variable immunodeficiency (CVID;N=45), isolated or undefined antibody deficiencies (N=108) and phagocyte defects (N=3)) and 73 controls. SARS-CoV-2-specific IgG titers, neutralizing antibodies, and T-cell responses were evaluated. One year after the start of the COVID-19 vaccination program, 334 study participants (239 IEI patients and 95 controls) completed a questionnaire to supplement their clinical data focusing on SARS-CoV-2 infections.

**Results:**

After booster vaccination, S-specific IgG titers increased in all COVID-19 naive IEI cohorts and controls, when compared to titers at 6 months after the priming regimen. The fold-increases did not differ between controls and IEI cohorts. SARS-CoV-2-specific T-cell responses also increased equally in all cohorts after booster vaccination compared to 6 months after the priming regimen. Most SARS-CoV-2 infections during the study period occurred in the period when the Omicron variant had become dominant. The clinical course of these infections was mild, although IEI patients experienced more frequent fever and dyspnea compared to controls and their symptoms persisted longer.

**Conclusion:**

Our study demonstrates that mRNA-based booster vaccination induces robust recall of memory B-cell and T-cell responses in most IEI patients. One-year clinical follow-up demonstrated that SARS-CoV-2 infections in IEI patients were mild. Given our results, we support booster campaigns with newer variant-specific COVID-19 booster vaccines to IEI patients with milder phenotypes.

## Introduction

Inborn errors of immunity (IEI), commonly referred to as primary immunodeficiencies (PID), are a diverse group of congenital disorders affecting single or multiple components of the immune system. IEI result in increased susceptibility to infections, and sometimes autoimmune complications, autoinflammatory diseases, allergies and an increased risk for malignancies. In many IEI disturbed or absent responses to vaccination are found. During the COVID-19 pandemic, patients with IEI were prioritized in the Dutch COVID-19 vaccination program to receive 2 doses of an mRNA-based COVID-19 vaccine (mRNA-1273). Multiple studies have investigated the immunogenicity of COVID-19 vaccines in these patients. We and others found that in patients with primary antibody deficiencies an overall serologic response of 72% was observed, ranging from 0% in X-linked agammaglobulinemia (XLA) patients, 52-81% in common variable immunodeficiency (CVID) patients, to 100% in specific polysaccharide antibody deficiency (SPAD) patients (1–3). In patients with combined immunodeficiencies (CID), variable serological responses have been described, ranging from 0 to 100%, although the numbers of studied patients were low and clinical phenotypes heterogeneous (1, 2, 4, 5). In addition, SARS-CoV-2 specific T-cell responses in IEI patients are reported to be robust and comparable to those in controls (1, 6). Although response rates after vaccination were promising, lower levels of neutralizing antibodies were detected in IEI patients when compared to controls, which raised questions about the long-term protection and the need for booster vaccinations (1–3).

Recently, we reported the six-month immunogenicity of the mRNA-1273 COVID-19 vaccine in our cohort of Dutch IEI patients (7). Binding and functional antibody titers significantly declined at six months after the second vaccination in both IEI patients and controls, with no differences in decay rates. However, antibody titers at 28 days after vaccination in patients with CID and CVID were lower when compared to controls, and antibody titers dropped below the responder cut-off in these patients more frequently at six months after completion of the priming regimen. Moreover, most CVID patients that did not respond to the initial regimen of two mRNA-1273 COVID-19 vaccines, did not respond to a third vaccination either (7, 8).

In addition to declining antibody titers after the priming regimen, the Omicron variant, which emerged in late 2021, showed a sharp reduction in sensitivity to neutralizing antibodies, leading to reduced or absent neutralization of this variant in healthy individuals (9). Booster vaccination partially restored this neutralizing capacity against Omicron (9–13). As a consequence, adults, including IEI patients, were advised to receive booster vaccinations. Although boosters enhance vaccine effectiveness, their effects wane over time, leading to more breakthrough infections (14). A Danish study found a correlation between higher Spike (S)-specific antibody titers and a reduced risk of breakthrough infections for the Delta variant, but this correlation was not demonstrated for the Omicron variant (15).

Various studies have described the effects of boosters in heterogeneous cohorts of IEI patients, showing an increase in antibody titers and/or neutralizing capacities (8, 16). However, no study has specifically analyzed the effects of boosters in different subgroups of IEI patients, which is crucial to determine which specific patients would benefit most from additional boosters. This knowledge is essential for policy-making to allow for optimal (booster) vaccination strategies in these different cohorts. While clinical data on breakthrough infections in IEI patients after the initial vaccination regimen is available, limited data exists on breakthrough infections following booster vaccination (17, 18). A large cohort study conducted in the United Kingdom demonstrated an increased risk of hospitalization and mortality after breakthrough infections following a mRNA booster dose in individuals with an immunodeficiency compared to healthy subjects. However, the study did not specifically examine this risk in IEI patients (19). Therefore, in the present study, we investigated SARS-CoV-2-specific antibody and T-cell responses after the administration of mRNA-based boosters in 171 IEI patients and 73 controls. Patients were categorized into cohorts based on different types of immunodeficiencies. These cohorts include XLA, CVID, CID, isolated antibody deficiencies (IgG subclass deficiency ± IgA deficiency (IgG), SPAD, phagocyte defects, and undefined antibody deficiencies. Additionally, we measured neutralizing antibodies 8 weeks after booster vaccination in participants who experienced breakthrough infections after booster vaccination and compared them to those who did not have breakthrough infections. Finally, we examined the clinical outcomes of our IEI cohort for up to one year after the start of the Dutch national vaccination campaign.

## Methods

### Ethical statement

The Vaccination Against COvid in Primary Immune Deficiencies (VACOPID) study is a prospective, controlled, multicenter research initiative carried out in seven academic hospitals in the Netherlands, involving patients with IEI. The study adheres to the principles of the Declaration of Helsinki and has received approval from the Dutch Central Committee on Research Involving Human Subjects (CCMO, NL7647.078.21, EudraCT number 2021-000515-24), the Medical Research Ethics Committee from Erasmus University Medical Center (MEC-2021-0050) and the local review boards of all other participating centers. Written informed consent was provided by all participants before inclusion.

### Study participants and design

**Figure 1:**
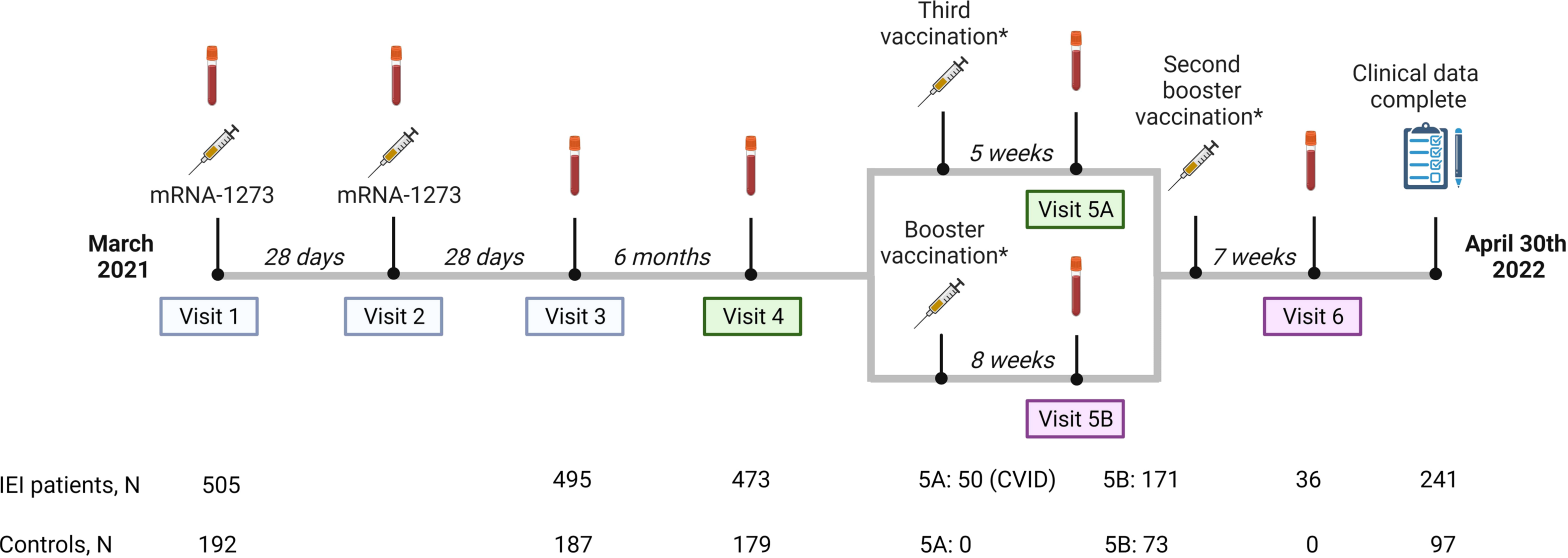
Study design. Study design including study visits, number of participants per study visit and median intervals between study visits or between vaccination and study visits. Study visit 5 is divided into part A and B. Part A are participants receiving a third vaccination. In our study, these participants are CVID patients who use immunosuppressive drugs, or in specific individual cases when a medical specialist had reasonable arguments to make an exception to the aforementioned indications, based on proven or assumed non-response. Part B are participants who received a regular booster vaccination. Results of study visits 1, 2 and 3, 4 and 5A were published previously (blue and green panels). Results of study visit 5B and 6 (pink panels) are included in this study.*The mRNA-1273 (Moderna)- and the BNT162b2 (Pfizer) vaccines were used as third vaccination or booster vaccination based on local availability and personal preferences and were administered at public vaccination sites.

A total of 505 patients with IEI and 192 non-IEI controls were initially included and stratified into different cohorts (**Table 1**, **Figure 1**). The study design and criteria for inclusion/exclusion are described in detail in the **Supplementary Material**. The XLA, CID and CVID cohorts are characterized by more severe clinical phenotypes. Thereby, within the CVID cohort, patients may also experience auto-immune, granulomatous, lymphoproliferative and/or oncological complications. Isolated IgG subclass deficiency ± IgA deficiency and SPAD are clinically similar cohorts with a milder clinical phenotype, and were studied as one group. The undefined antibody deficiency cohort included patients with primary hypogammaglobulinaemia and preserved T-cellular immunity who do not meet the diagnostic criteria for any other primary antibody deficiencies. SARS-CoV-2 infections reported by the participants were also tracked throughout the study. At the beginning of the study, all study participants received two doses of the mRNA-1273 COVID-19 vaccine, administered with a 28-day interval, in accordance with the Dutch COVID-19 vaccination program. Results of the immune responses at 28 days- and six months following the second mRNA-1273 COVID-19 vaccine have been previously published (1, 7).

**Table 1:** Baseline characteristics of VACOPID participants that donated a blood sample after booster vaccination.

		Controls	X-linked agamma-globul-inemia (XLA)	Combined Immunode-ficiency (CID)	Common Variable Immunodeficiency (CVID)	Isolated IgG subclass deficiency + Specific polysaccharide antibody deficiency (SPAD)	Phagocyte defects	Undefined antibody deficiency	Total IEI	P-values (Total IEI vs. control)
Baseline characteristics of participants that did not contract a SARS-CoV-2 infection before blood sample donation after booster vaccination										
n		54	7	4	29	79	2	4	125	
Male, n(%)		26 (48%)	7 (100%)	2 (50%)	12 (41%)	29 (37%)	2 (100%)	2 (50%)	54 (43%)	.62^A^
Median age (IQR)		57 [27-73]	47 [27-63]	34 [22-55]	55 [19-79]	60 [22-77]	36 [19-53]	36 [31-66]	57 [19-79]	.79^B^
Booster vaccine										
Moderna		31	3	1	12	45	1	2	64	
Pfizer		20	3	3	15	31	1	2	55	
mRNA booster, not further specified		3	1		2	3			6	
Immunoglobulin replacement therapy, n (%)		0	7 (100%)	1 (25%)	26 (90%)	42 (53%)	0	4 (100%)	80 (64%)	
IEI with ≥ 1 non-infectious complication, n (%)		0	2 (29%)	2 (50%)	8 (28%)	21 (27%)	1 (50%)	1 (25%)	35 (28%)	
Immunosuppressive medication used in past 2 years and during the study										
Prednisone/other corticosteroid treatment		1	1		9	22			32	
Azathioprine					1	1			2	
Anti-TNF-a						1			1	
Hydroxychloroquine					1	2			3	
Mycophenolate mofetil						1			1	
Other DMARDs						1			1	
Methotrexate					1	2			3	
Calcineurin inhibitors						1			1	
JAK inhibitors				1					1	
SARS-Cov-2 infections in participants that donated a blood sample after booster vaccination										
None		41	6	4	21	64	2	4	101	
Before start of the study		2	1	0	3	7	0	2	13	
Between second vaccination and booster vaccination		3	0	0	2	4	0	0	6	
Between booster vaccination and blood sample collection		12	2	0	9	9	0	1	21	
Between blood sample collection after booster vaccination and end of study		13	1	0	8	15	0	0	24	

A, Fisher exact test; B, Wilcoxon rank-sum test; TNF, tumor necrosis factor; DMARD, disease modifying anti-rheumatic drugs; JAK, Janus kinase.

A third mRNA-based COVID-19 vaccine was recommended by the Dutch healthcare authorities to patients with CID or CVID who use immunosuppressive drugs, or in specific individual cases, based on proven or assumed non-response (20). These so-called third vaccinations were mainly administered in October 2021 (**Figure 1**, visit 5A). Alongside this third dose, a booster vaccination campaign was launched, recommending mRNA-based boosters based on the ancestral SARS-Cov-2 strain for adults who received their last COVID-19 vaccination and/or had a SARS-CoV2 infection more than three months ago. Both controls and IEI patients who were not eligible for a third vaccination, and IEI patients who received a third vaccination more than three months ago were eligible for this booster vaccination. Most participants received their booster vaccination in December 2021 and January 2022. The mRNA-1273 (Moderna) and the BNT162b2 (Pfizer) vaccines were used as boosters based on local availability and personal preferences and were administered at public vaccination sites (**Figure 1**, visit 5B).

An amendment to the original study protocol was approved by the Medical Research Ethics Committee from Erasmus University Medical Center. With this amendment the immune responses of mRNA-based COVID-19 booster vaccination in our patient cohort could be studied. Participants of six academic centers were invited to donate additional blood samples four to ten weeks after booster vaccination. 171 IEI patients and 73 controls responded to this invitation. Three months after the third vaccination or booster, IEI patients could receive another booster vaccination according to the Dutch vaccination program. Blood was collected from 36 IEI patients four to ten weeks after receiving this 4th dose (**Figure 1**, visit 6). The study period ran until April 30^th^ 2022. At the end of the study, all participants that were initially included were asked to complete a questionnaire to supplement their clinical data, regardless of whether they had completed all study visits. This request was fulfilled by 338 participants (241 IEI patients and 97 controls).

### Measurement of humoral and cellular immune responses

The assays to evaluate humoral and cellular immune responses are described more extensively in the **Supplementary Material**. Briefly, the quantitative Luminex assay was used to measure S-specific IgG and nucleocapsid (N) specific IgG, with results expressed as international Binding Antibody Units per mL (BAU/ml) (21, 22). Participants with S-specific IgG above 44.8 BAU/ml were considered seropositive (23). Nucleocapsid (N)-specific IgG antibodies were analyzed to identify participants who contracted COVID-19 before or during the study. We selected a cutoff value with 100% specificity, measured in pre-pandemic sera from healthy donors, to minimize the possibility of false-positive results due to administration of (low levels of) N-specific antibodies in IGRT (23, 24). Therefore, an infection with SARS-CoV-2 was defined as a history of a positive PCR test, positive antigen test, and/or an N-specific IgG titer above 42.2 BAU/ml. We tested samples for the presence of neutralizing antibodies from 104 participants (32 controls, 19 CVID, 53 IgG/SPAD) whose clinical information was completed and who had not experienced COVID-19 until blood collection after booster vaccination. We compared serum neutralization capacity between participants who contracted COVID-19 after blood sample collection and those who remained uninfected, using a pseudovirus system targeting ancestral SARS-CoV-2 and Omicron subvariants BA.1 and BA.2. Neutralization titers were expressed as International Units per mL (IU/ml) (25, 26). The WHO International Standard for anti-SARS-CoV-2 immunoglobulin (NIBSC 20/136) was used to normalize the IgG and neutralizing antibody titers. T-cell responses were assessed in samples obtained from the university hospitals of Leiden and Rotterdam using an Interferon-gamma (IFN-ɣ) release assay (IGRA, QIAGEN) including a peptide pool that covers the S protein (Ag2). The results of the IGRA were quantified in IU IFN-ɣ/ml after subtraction of the negative control value.

### Statistical analysis

Categorical variables are displayed as numbers and percentages and analyzed with Fisher’s exact test. Continuous variables are presented as median ± interquartile range (IQR). The time interval between booster administration and blood collection was corrected for outliers. Outliers were defined as below the first quartile minus 1.5 times the IQR or above the third quartile plus 1.5 times the IQR. Fold changes are displayed as the median of individual fold changes. Results of the immunological assays were displayed in figures and text with geometric means, medians, and (interquartile) ranges. The Wilcoxon rank-sum test was used to analyze continuous variables and the Wilcoxon signed rank test was used to analyze paired data. Log-rank tests were used to compare breakthrough infections after booster vaccination. P-values below 0.05 were considered statistically significant.

### Software

Study data were collected in an online electronic data capture system (Castor^©^, Amsterdam, the Netherlands), compliant to the General Data Protection Regulation (GDPR). R studio was used for statistical analyses. Graphs were prepared with GraphPad PRISM, version 9.1.2 (San Diego, CA, USA).

## Results

Blood samples were collected from a total of 244 participants (171 IEI and 73 controls) who received a booster vaccination (**Figure 1**). The median time between the second vaccination of the priming regimen and the booster vaccination was 258 days (IQR 244-267), the median time between the blood sample taken 6 months after second vaccination and booster vaccination was 74 days (IQR 61-85) and the median time between booster administration and blood sample collection was 59 days (IQR 49-67). None of these participants had previously received a third vaccine. Eight participants (2 controls, 6 IEI) were excluded from analysis due to outlying intervals between booster administration and blood sample collection (>94 days). Forty IEI patients and 17 controls who had contracted COVID-19 before- or during the study were analyzed separately. COVID-19 was diagnosed by PRC or antigen test in 50 participants (13 controls, 37 IEI), while 7 (4 controls, 3 IEI) were diagnosed by anti-N antibodies above 42.2 BAU/ml. After excluding the participants who had contracted COVID-19, 125 IEI patients and 54 controls remained eligible for the main analysis of the booster vaccination study. Their baseline characteristics are displayed in **Table 1**. As the numbers of patients with phagocyte defects and undefined antibody deficiencies were low, findings regarding these cohorts are reported in detail in the **Supplementary Material** (**Supplementary Figure 1**).

### Booster vaccination increases SARS-CoV-2 S-specific IgG titers in IEI patients

**Figure 2:**
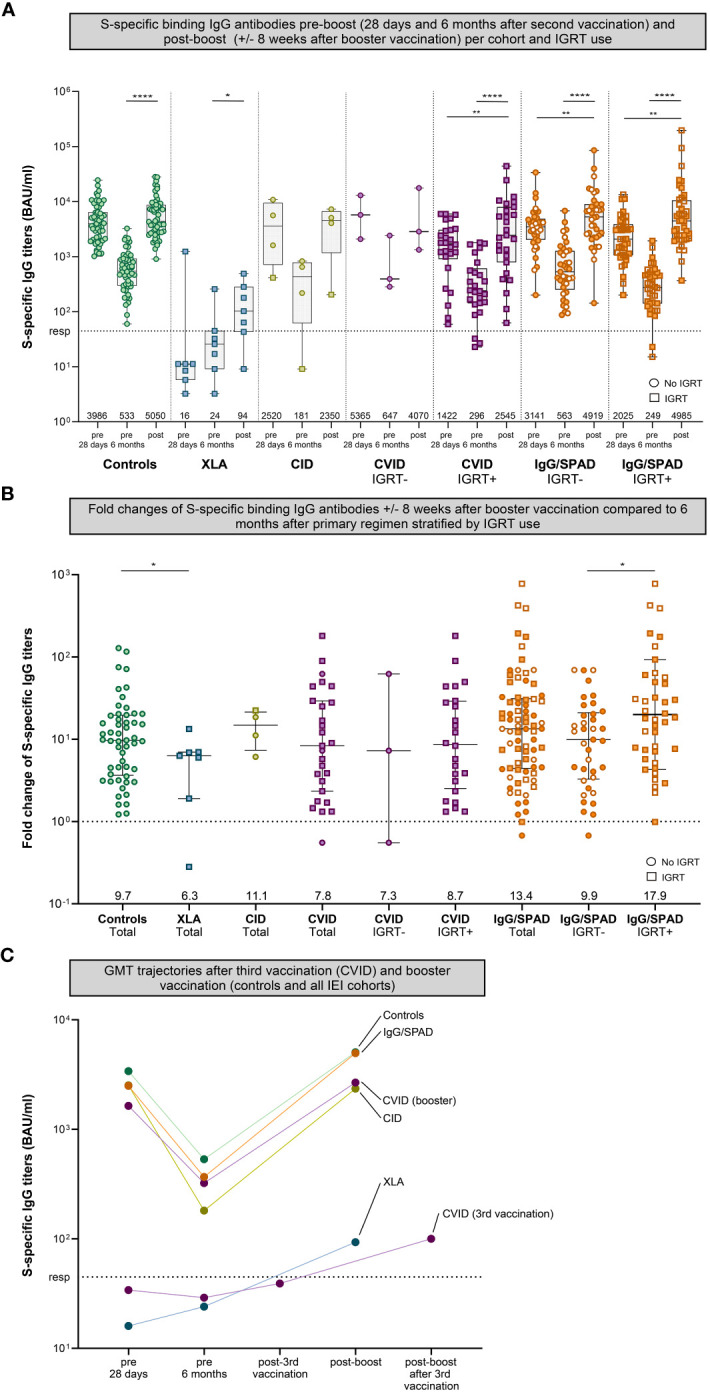
S-specific IgG pre- (28 days and six months after second vaccination) and post-boost (+/- 8 weeks after booster vaccination). **(A)** S-specific IgG measured by Luminex for controls and different cohorts of inborn errors of immunity (IEI) patients 28 days after second vaccination, six months after the second vaccination, and eight weeks after booster vaccination. The number of participants per cohort correspond to **Table 1**. The CVID and IgG cohort were stratified based on IGRT use. Results are expressed in binding antibody units per milliliter (BAU/mL). The dotted line is the pre-defined responder cut-off (resp). Data in panel A are presented in box-and-whisker plots. The horizontal lines of the box-and-whisker plots indicate the median, the bounds of the boxes indicate the interquartile range, and the whiskers indicate the range. All datapoints are shown. The numbers below the box-and-whisker plots indicate the geometric mean titers (GMT) per time point. Participants not using immunoglobulin replacement therapy (IGRT) are shown as circles, participants using IGRT are shown as squares. IgG titers were compared per cohort using the Wilcoxon paired signed rank test. The SPAD cohort is indicated with white symbols (with orange borders) while the IgG cohort is indicated with orange symbols. **(B)** Fold changes of IgG antibodies post-boost (+/- 8 weeks after booster vaccination) compared to 6 months after primary regimen of each cohort in total plus the fold changes the CVID and IgG/SPAD cohort stratified by IGRT use. Data in panel B are presented in scatter dot plots. The horizontal lines indicate the median, the whiskers indicate the interquartile range. All data points are shown. The dashed line represents a fold change of 1, where the titer at 6 months after primary regimen is equal to the titer after booster. All data points above the dashed line represents a fold increase, all data points below the dashed line a fold decrease. The numbers below indicate the median fold change. Fold changes were compared per cohort using the Wilcoxon rank-sum test. **(C)** Trajectories of the GMTs after third vaccination (CVID) and booster vaccination (all cohorts). The results of CVID participants receiving a third vaccination have also been published previously. A subset of these CVID participants received a booster after their third vaccination (fourth dose in total, named post-boost after 3^rd^ vaccination on the x-axis). The results after booster vaccination of the CVID cohort and the other cohorts were computed only on those who donated blood after booster vaccination. Color coding is the same in all figures. S, Spike; XLA, X-linked agammaglobulinemia; CID, Combined Immunodeficiency; CVID, Common Variable Immunodeficiency; IgG, Isolated IgG subclass deficiency ± IgA deficiency; SPAD, Specific polysaccharide antibody deficiency; * = P<.05, ** = P<.01, *** = P<.001, **** = P<.0001.

To determine to what extent SARS-CoV-2 S-specific antibody titers increase after booster vaccination, S-specific IgG titers at 6 months after the second vaccination were compared with S-specific IgG titers 6-10 weeks after booster vaccination. No differences were observed in responses following either a mRNA-1273 (Moderna) or BNT162b2 (Pfizer) booster (**Supplementary Figure 2**); the results hereafter represent the pooled outcomes. Booster vaccination increased SARS-CoV-2 S-specific IgG titers in all cohorts when compared to the titers six months after the second vaccination. The geometric mean titer (GMT) of S-specific IgG in the control cohort increased 9.7-fold, from 533 to 5050 BAU/ml (P<.0001) (**Figure 2**). The GMT’s of the CVID and IgG/SPAD cohorts (in total) also increased significantly (CVID: 7.8 fold from 322 BAU/ml to 2670 BAU/ml (P<.0001), IgG/SPAD: 13.4-fold from 367 to 4950 BAU/ml (P<.0001)). When stratifying these cohorts based on the use of immunoglobulin replacement therapy (IGRT), S-specific IgG still increased significantly in CVID patients on IGRT (8.7 fold from 296 BAU/ml to 2545 BAU.ml (P<.0001)) and IgG/SPAD patients with and without IGRT use (with IGRT: 17.9 fold from 249 BAU/ml to 4985 BAU/ml (P<.0001), without IGRT: 9.9 fold from 563 BAU/ml to 4919 BAU/ml (P<.0001)). No significant increase was observed in the smaller cohorts, consisting of the CVID cohort without use of IGRT (N=3) and the CVID cohort (N=4)(7.3 fold from 647 BAU/ml to 4070 BAU/ml (P=.50), and 14.8-fold from 181 to 2350 BAU/ml (P=.13), respectively) (**Figure 2**). Although the geometric mean titer (GMT) of S-specific IgG in the XLA cohort increased significantly, this titer is still very low (6.3-fold from 24 to 94 BAU/ml (P=.03)) and this effect should be contributed to IGRT. The GMT’s of S-specific IgG after booster vaccination did not differ between patients in the CVID and IgG/SPAD cohort who received IGRT and those who did not (P=.65 and P=.77, respectively) (**Figure 2A**). However, fold changes of the IgG/SPAD patients who use IGRT were significantly higher compared to the fold changes of IgG/SPAD patients who did not use IGRT (P=.03) (**Figure 2B**). Fold changes did not differ between (total) IEI cohorts and controls, except for the XLA cohort (P=.02). Trajectories of the GMTs of the different cohorts are shown in **Figure 2C**. This figure also includes previously reported results of CVID participants that received a third vaccination (instead of a booster vaccination) (7).

### COVID-19 does not result in higher SARS-CoV-2 S-specific IgG titers after booster vaccination

In total, 15 controls and 40 IEI patients who donated a blood sample after receiving a booster vaccination contracted COVID-19 before this blood donation. The majority of infections occurred before the first vaccination (N=15, 27.3%) or between the booster vaccination and the collection of the blood sample (N=30, 55%) (**Table 1**). GMT of participants who contracted COVID-19 before or during the study were similar to those who did not contract COVID-19 (**Supplementary Figure 3**).

### Neutralizing antibodies in the IgG/SPAD cohort as correlate of protection against breakthrough infections

Neutralizing antibodies were compared between participants who contracted COVID-19 after blood sample collection and those who remained uninfected, focusing on our largest cohorts (controls (N=32), CVID (N=19) and IgG/SPAD (N=53)). The follow-up duration after blood sample collection was comparable between participants who did and who did not contract COVID-19 (median 67 days (IQR 62-72) and 66 days (IQR 58-72) respectively, P=.18). Significantly lower neutralizing antibody titers were found in IgG/SPAD patients who developed COVID-19 shortly after blood sample collection (ancestral: P=.0021, BA.1: P=.0012, BA.2: P=.0041) (**Supplementary Figure 4**). Similarly, IgG/SPAD participants with relatively low titers of neutralizing antibodies against the ancestral virus, BA.1, and BA.2 (below the median of 283 IU/ml, 82.5 IU/ml, and 136 IU/ml respectively) had a significantly higher risk of contracting a breakthrough infection during the initial months after blood sample collection (Ancestral: hazard ratio 3.5, 95% confidence interval [1.02-12.17], P=.047. BA.1 and BA.2: hazard ratio 5.2, 95% confidence interval [1.50-17.92], P=.0093). The same trend was seen when comparing binding antibodies, with anti-S antibodies being significantly lower in IgG/SPAD participants who developed COVID-19 shortly after blood sample collection (IgG/SPAD: P=0.006, controls: P=.30, CVID: P=.18).

### Booster vaccination increases SARS-CoV-2 specific T-cell responses

**Figure 3:**
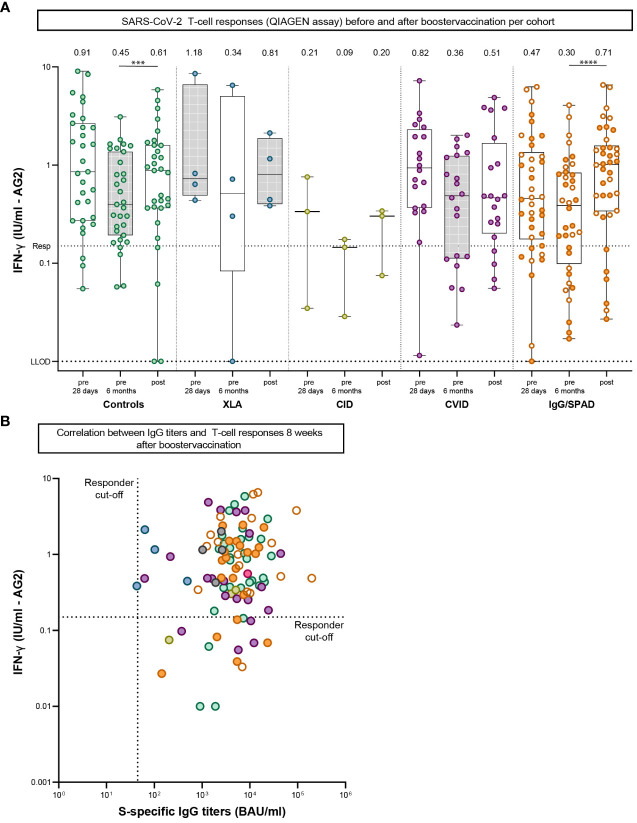
SARS-CoV-2-specific T-cell responses pre-boost (28 days and six months after second vaccination) and post-boost (+/- 8 weeks after booster vaccination). **(A)** SARS-CoV-2-specific T-cell responses measured by an interferon γ (IFN-γ) release assay (QIAGEN) after stimulation of whole blood 28 days and six months after second vaccination and eight weeks after booster vaccination. Lower limit of detection (LLOD) is.01 IU/ml and responder cut off (resp) is.15 IU/ml. Results are expressed as international units/milliliter (IU/mL). The dotted line is the pre-defined responder cut-off (resp). Data is presented in box-and-whisker plots. The horizontal lines of the box-and-whisker plots indicate the median, the bounds of the boxes indicate the interquartile range, and the whiskers indicate the range. All datapoints are shown. The numbers above the box-and-whisker plots indicate the geometric mean titer (GMT). Within each cohort, IFN-γ levels at 28 days and six months were compared using Wilcoxon paired signed rank test. The SPAD cohort is indicated with white symbols while the IgG cohort is indicated with orange symbols. **(B)** Correlation between IgG titers and T-cell responses 8 weeks after booster vaccination. The dotted horizontal line is the responder cut-off of the QIAGEN interferon-gamma release assay (0.15 IU/mL). The dotted vertical line is the responder cut-off of the Luminex assay (44.8 BAU/ml). Color coding is the same in all figures. XLA, X-linked agammaglobulinemia; CID, Combined Immunodeficiency; CVID, Common Variable Immunodeficiency; IgG, Isolated IgG subclass deficiency ± IgA deficiency; SPAD, Specific polysaccharide antibody deficiency; *** = P<.001, **** = P<.0001.

SARS-CoV-2-specific T-cell responses after booster vaccination were assessed in samples collected from two study sites using the QIAGEN assay (**Figure 3A**). In this assay, IFN-γ levels after booster vaccination increased significantly from 0.45 IU/ml to 0.61 IU/ml in the control cohort (P<.001) and from 0.30 IU/ml to 0.71 IU/ml in the IgG/SPAD cohort (P<.0001) compared to the IFN-γ levels 6 months after the primary regimen. No significant increase was observed in the other (smaller) cohorts (XLA: 0.34 to 0.81 IU/ml (P=.88), CID: 0.09 to 0.20 IU/ml (P=.50), CVID: 0.36 to 0.51 IU/ml (P=.08). IFN-γ levels did not increase after booster vaccination compared to the initial response, and IFN-γ levels after booster were not statistically different between controls and IEI cohorts. Of the 101 participants analyzed, 15 (9%) did not have IFN-γ levels above the responder cut-off of 0.15 IU/ml (4 controls, 1 CID, 4 CVID and 6 IgG/SPAD), but all these T-cell non-responders had SARS-CoV-2 S-specific IgG titers above the cut-off level (44.8 IU/ml) (**Figure 3B**).

### CVID cohort

**Figure 4:**
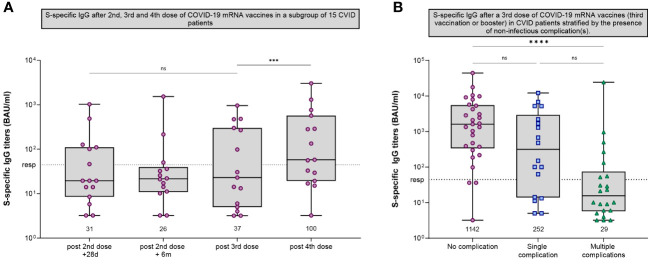
SARS-CoV-2-specific IgG in CVID participants. **(A)** S-specific IgG measured by Luminex for CVID patients that received a third dose and a (fourth) booster dose after the initial regimen of 2 mRNA-1273 COVID-19 vaccines. S-specific IgG was measured 28 days after second vaccination, six months after the second vaccination, five weeks after third vaccination and eight weeks after booster vaccination. **(B)** S-specific IgG measured by Luminex for CVID patients after a third dose of a mRNA COVID-19 vaccine (either a third vaccination of a booster dose) stratified by the presence of non-infectious complications. The following non-infectious complications were defined: Autoimmune cytopenia, organ specific autoimmunity, systemic autoimmunity, enteropathy, malignancy, lymphoproliferative diseases, granulomatous lymphocytic interstitial lung disease (GLILD), and other granulomatous diseases. **(A, B)** Results are expressed in binding antibody units per milliliter (BAU/mL). The dotted line is the pre-defined responder cut-off. Data in panels A and B are presented in box-and-whisker plots. The horizontal lines of the box-and-whisker plots indicate the median, the bounds of the boxes indicate the interquartile range, and the whiskers indicate the range. All datapoints are shown. The numbers below the box-and-whisker plots indicate the geometric mean titer (GMT) per timepoint. IgG titers were compared per cohort using the Wilcoxon paired signed rank test. CVID, Common Variable Immunodeficiency; *** = P<.001, **** = P<.0001; ns = not significant.

Clinically, CVID patients are known to experience recurrent (bacterial) infections with varying severity. A number of CVID patients show additional autoimmune, granulomatous, lymphoproliferative and/or oncological complications (27). As reported previously, fifty CVID patients of our cohort received a third vaccination (7). Fifteen of them also donated blood samples after receiving a fourth dose (administered at least three months after the third vaccination). After this fourth dose, the GMT of anti-S IgG increased from 37 BAU/ml to 100 BAU/ml (P=.0006) (**Figures 2C**, **4A**). Although this was a significant increase, the GMT is still considered low compared to the trajectories of the other cohorts. Fourteen of these participants received IGRT, which may also explain the increase due to increasing levels of anti-S antibodies in those preparations at the time (24). Previously, we found that the presence of a noninfectious complication with or without the use of immunosuppressive medication was negatively associated with response after two mRNA-1273 vaccinations. This association persisted after administration of a third dose of a mRNA COVID-19 vaccine (third vaccination or booster) (**Figure 4B**) (**Supplementary Table 1**).

### Clinical outcomes one year after primary vaccination regimen

After one year of follow-up, 243 study participants had contracted at least one SARS-CoV-2 infection (66 controls, 178 IEI patients) (**Table 2**). Reinfections with SARS-CoV-2 were reported in 5 controls (7%) and 20 IEI patients (10%). Eleven participants were admitted to a hospital because of COVID-19. Main reasons for admission included symptomatic illness by COVID-19 and/or admission to day clinics for (experimental) monoclonal antibody treatment (**Table 3**). The binding antibody titers, measured 28 days after second vaccination, were not different between CVID and IgG/SPAD patients who were hospitalized due to COVID-19 related illness and patients from the same cohorts who were not hospitalized (P=.48 and P=.45 respectively). The number of hospitalized patients in which T-cell response was evaluated was too low to correlate T-cell responses with risk of hospitalization due to COVID-19 (data not shown). Clinical data on the occurrence of COVID-19 has been completed up to the study endpoint in 338 participants (241 patients with IEI and 97 controls) (**Table 2**). Of these 338 participants, two CVID patients and one CID patient died during the study. Their causes of death were unrelated to COVID-19. Of the remaining 335 participants, 130 infections occurred in the IEI patients and 50 in the control cohort, resulting in similar infection rates (54% and 52% respectively). Most participants were infected after the Omicron lineages became dominant. COVID-19 related symptoms including cough, dyspnea, fever, nasal congestion, throat pain, and the duration of symptoms were evaluated by a COVID-19 questionnaire. IEI patients experienced significantly more frequent fever and dyspnea compared to controls (40% and 37% in IEI patients, 16% and 8% in controls respectively, P=.024 and P=.002). Additionally, symptom duration was significantly longer (IEI median 8 days [IQR 5-14], controls median 5 days [IQR 3-7], P<.0001) (**Table 2**). Subsequent regression analyses revealed that variables including gender, age, underlying disease, use of IGRT and/or immunosuppressive medication were not able to explain the observed difference in symptom duration (data not shown).

**Table 2:** Total SARS-CoV-2 infections and detailed information on SARS-CoV-2 infections.

		Controls N=188	X-linked agammaglobulinemia(XLA) N=21	Combined Immunodeficiency (CID) N=24	Common Variable Immunodeficiency (CVID) N=210	Isolated IgG subclass deficiency ± IgA deficiency N=129	Specific polysaccharide antibody deficiency (SPAD) N=64	Undefined antibody deficiency N=22	Phagocyte defects N=16	Total IEI N=486	P-values (controls vs Total IEI)
*Participants with COVID-19 infections (% of total inclusions)*		*66 (35)*	*5 (24)*	*5 (21)*	*85 (40)*	*48 (37)*	*24 (38)*	*7 (32%)*	*4 (25)*	178 (37)	0.72
Reinfections		5	0	1	9	7	0	2	1	20	.48 ^A^
Hospital admissions		0	0	0	9	2	0	0	0	11	
Number of complete study records		97	10	15	93	67	41	9	6	241	.78 ^A^
SARS-CoV-2 infections of complete study records (% of complete records)*:		50 (52)	2 (20)	6 (40)	65 (70)	33 (49)	15 (37)	4 (44)	5 (83)	130 (54)	.82 ^A^
Ancestral		5	0	0	4	2	0	1	1	8	
Alpha (B.1.1.7)		0	0	1	1	1	0	0	0	3	
Delta (B.1.617.2)		2	0	1	4	3	1	0	1	10	
Omicron (B.1.1.529)		38	2	4	52	24	13	1	3	99	
Unknown**		5	0	0	4	3	1	2	0	10	
Median duration of symptoms (days)		5	3	6	7	7	12	10	NA	8	**<.0001** ^B^
Symptoms:											
Cough		19	0	2	30	18	8	2	0	60	.65 ^A^
Dyspnea		4	0	0	22	17	7	2	0	48	**.002** ^A^
Fever		8	0	2	23	17	8	1	1	52	**.024** ^A^
Nasal congestion		23	1	1	29	20	6	0	0	57	.88 ^A^
Throat pain		17	1	1	20	16	7	1	0	46	1 ^A^

A: Fisher exact test. B: Wilcoxon rank-sum test. *Based on which variant was dominant at the time of infection. **Exact date of infection missing.

**Table 3:** Hospital admissions of IEI patients related to COVID-19.

	Cohort	Reason admission	Dominant strain during admission	Treatment
1	CVID	Illness caused by COVID-19	Alpha (B.1.1.7)	Convalescent plasma treatment Corticosteroid treatment
2	CVID	Admission at day clinic for experimental treatment	Delta (B.1.617.2)	Casirivimab/Imdevimab
3	CVID	Admission at day clinic for monoclonal antibody treatment	Omicron (B.1.1.529)	Sotrovimab
4	IgG	Illness caused by COVID-19	Omicron (B.1.1.529)	Corticosteroid treatment
5	CVID	Illness caused by COVID-19	Omicron (B.1.1.529)	Albuterol/ipratropium combination Antibiotics for prevention of secondary infection
6	CVID	Admission at day clinic for monoclonal antibody treatment	Omicron (B.1.1.529)	Sotrovimab
7	CVID	Urosepsis. No clinical symptoms of COVID-19, was tested because of contact tracing	Omicron (B.1.1.529)	Antibiotic treatment for urosepsis
8	CVID	Illness caused by COVID-19	Omicron (B.1.1.529)	Corticosteroid treatment Antibiotics for prevention of secondary infection
9	CVID	Illness caused by COVID-19	Omicron (B.1.1.529)	Medical observation
10	IgG	Illness caused by COVID-19	Omicron (B.1.1.529	Short-term observation because of pregnancy
11	CVID	Illness caused by COVID-19	Omicron (B.1.1.529)	Tocilizumab Corticosteroid treatment

## Discussion

This study investigated the effect of mRNA-based COVID-19 booster vaccination in a group of 171 IEI patients. Booster vaccination increased S-specific IgG titers in all cohorts when compared to the titers six months after the priming vaccination regimen. In addition, we found that lower neutralizing antibody titers after booster vaccination in the IgG/SPAD cohort were predictive of developing COVID-19 shortly thereafter. Clinical evaluation of the IEI cohort one year after primary vaccination showed that incidence of COVID-19 was comparable between IEI patients (54%) and controls (52%) and only 11 out of 177 IEI patients that contracted COVID-19 were admitted to hospital, none of whom required admission to an intensive care unit.

The increase in antibody titers after booster vaccination in patients with IEI suggests that the first two mRNA-1273 vaccinations induced immunological memory, which is also known from previous studies in non-IEI cohorts (28). Specifically, the IEI cohorts with milder clinical phenotypes, such as the IgG subclass deficiency ± IgA deficiency (IgG) and specific polysaccharide antibody deficiency (SPAD), show similar trajectories of antibody titers compared to the control cohort. The fold increase of S-specific IgG titers following booster vaccination, in comparison to titers 6 months after the primary vaccination regimen, were similar between control- and IEI cohorts (except XLA). However, fold changes were significantly higher in IgG/SPAD participants that received IGRT compared to IgG/SPAD participants who did not. IgG/SPAD patients on IGRT generally have a more profound immunodeficiency and, as a result, exhibited significantly lower antibody titers 28 days and 6 months after the primary regimen compared to IgG/SPAD participant who did not receive IGRT. The findings of the higher fold changes after booster vaccination in these participants remain to be explained. Outliers in our dataset could contribute to this result, and the use of IGRT may explain some of the additional increase. However, we believe that the higher fold change cannot be fully attributed to the effect of IGRT, given the modest absolute increase in the XLA patients and the considerably lower fold change in the CVID patients receiving IGRT.

We found that IgG/SPAD patients with lower neutralizing antibody titers after booster vaccination had a higher risk of developing breakthrough infection during the following months. This is consistent with previous research investigating the importance of neutralizing antibodies as a correlate of protection against symptomatic SARS-CoV-2 infection (29, 30). In both the CVID and the control cohort, we did not find differences in titers between participants who did or did not experience breakthrough infections. This was possibly due to the lower sample sizes of these cohorts, but possible confounders such as differences in living situations or (protective) behavior could not be excluded.

Results from the CVID cohort, which constitutes the largest cohort in this study, are more difficult to interpret. This cohort encompasses a heterogeneous group of patients, and the vaccination regimens within this group varied. Some individuals received a third vaccination in addition to the priming vaccination regimen. These were primarily CVID patients who were using immunosuppressive drugs and/or exhibited proven or assumed non-response to the priming COVID-19 vaccination regimen (7). In addition to the limited impact of this third vaccination on S-specific IgG titers (7), our study revealed that an extra booster vaccination in this group (resulting in a total of four doses) did not result in a substantial increase of S-specific IgG antibodies either. The observed small increase in S-specific IgG antibodies likely reflected the general rise of these antibodies in IGRT preparations, as evidenced by the parallel trajectory of the increase of antibodies in the XLA cohort (**Figure 2B**). This finding is consistent with other studies that showed increasing titers of SARS-CoV-2-specific antibodies in IGRT preparations during our study period (16, 31–33). CVID patients who received a booster vaccination instead of a third dose generally exhibited a milder clinical disease and demonstrated an antibody trajectory comparable to that of controls.

The GMTs of the S-specific T-cell responses, as determined by measuring IFN-γ levels after stimulation of whole blood with peptides covering the S protein, increased after the booster vaccination compared with the levels observed at six months after the second vaccination. This confirms the presence of immunological memory, although the increase in T-cell responses was not statistically significant in all cohorts. This could be explained by the timing of blood collection. Previous studies demonstrated that T-cell responses after booster vaccination demonstrate the greatest increase within the first week post-vaccination, followed by a slight decline in the subsequent month (28, 34). Given that our assessment of T-cell responses was performed on average eight weeks after booster vaccination, it is plausible that our measured GMTs already started decreasing. In previous studies, cross-reactive CD4+ and CD8+ T-cells were detected after booster vaccination with monovalent mRNA-based vaccines, and these T-cells cross-reacted with the Omicron BA.1 variant (9, 35, 36). Notably, IFN-γ levels did not show significant differences between the control and IEI cohorts in our study. This suggests that IEI cohorts may also have developed cross-reactive T-cells following vaccination, which potentially protect them against severe disease after infection with an emerging SARS-CoV-2 variant. The advantageous role of COVID-19 mRNA vaccination in patients with IEIs was also recently demonstrated in another study that examined the breadth and epitope specificity of SARS-CoV-2-specific T cell receptor clonotypes (37).

In our study, IEI patients and controls had similar incidence rates of COVID-19. However, a Danish study with 313 CVID patients and 2504 controls found a higher incidence of SARS-CoV-2 among CVID patients. Despite this disparity, several similarities were observed between their study and ours. The majority of infections occurred after the emergence of the Omicron variant, and while the CVID group faced an increased risk of hospitalization due to COVID-19, their risk for mechanical ventilation and mortality did not differ significantly from the general population (38). On the other hand, other studies examining SARS-CoV-2 infections in IEI patients did identify an elevated case fatality rate, particularly in younger age groups, as well as an increased risk of intensive care admission (39). The prolonged duration of symptoms observed in IEI patients compared to controls in our study aligns with several other studies (39, 40).

This study has several limitations. Despite having 505 IEI patients and 192 controls at the beginning of this prospective cohort study, only 171 IEI and 73 controls donated blood following booster vaccination. Several reasons can be identified. One center did not complete the study. Moreover, despite being approached, many participants remained unresponsive. This could be due to lack of urgency because of the milder disease caused by the Omicron variant, which emerged around the time of the booster immunizations. However, it is impossible to rule out response bias, and the limited participants per cohort reduced the power of this study. Nevertheless, we were able to examine the immunogenicity of the booster vaccination in a relatively large number of IEI patients, in order to get a robust understanding of the “boostability” of IEI patients compared to controls. Furthermore, the assays we used in this study, for example the Luminex assay and the IGRA assay, are not able to identify whether the respective S-specific IgG antibodies or IFN-γ levels were derived from a memory B- and T-cells (recall response) or a *de novo* immune response. We assume that booster vaccination induces a recall response based on previous studies which have shown that S-specific IgG rapidly increases within 7 days after booster vaccination (28, 34). In addition, other studies demonstrated B- and T-cell memory by using assays that can specifically measure memory markers (9, 35, 41). In addition, the IGRA test used in this study focuses on the (S-specific) production of INF-γ and does not provide data on the quantity of SARS-CoV-2 specific T-cells. Finally, increasing concentrations of anti-S antibodies in IGRT could potentially have an effect on our results (32, 33). However, in our studies we found very low concentrations of antibodies in patients with XLA and we therefore consider the effects of IGRT to be limited at the time of our studies.

Our study implies that mRNA-based booster vaccination induces robust recall of memory B-cell and T-cell responses in IEI patients with a milder phenotype (CVID without non-infectious complications, SPAD, isolated antibody deficiencies, phagocyte defects, undefined antibody deficiencies). Although many participants were infected with the Omicron variant during the final months of the study period, the clinical outcomes remained favorable. The mild clinical presentation may potentially be explained by the broad cross-reactivity of T-cells, considering the limited neutralizing antibody response against Omicron following booster vaccination. Recently, a monovalent mRNA vaccine against the omicron XBB variant resulted in higher antibody titers compared to a bivalent variant (against the omicron XBB and omicron BA.4/BA.5 variants) in a non-IEI cohort and was recommended by the U.S. FDA for use in 2023-2024 immunizations (42). To our knowledge, there is currently no data available on variant-specific booster vaccination in IEI patients. However, we recommend these monovalent omicron XBB boosters to IEI patients with milder clinical phenotypes, given the favorable results observed thus far in our cohort (1, 7). It is more difficult to make a recommendation for severe IEI patient such as XLA and CVID with complicated disease. These patients are likely to have a persistently poor antibody after (variant-specific) booster vaccinations. Besides, the usefulness of giving virus-specific boosters for the aim of boosting (cross-reactive) T-cell responses is not clear yet. Rising levels of (neutralizing) antibodies in IGRT preparations offer perspective in these patients, although these preparations may have limited effect against newer variants because of the delay between plasma donations and their clinical use in IGRT preparations (24). Lastly, we argue that continued research into other therapies, such as antivirals, is needed to protect IEI patients from severe illness after contracting COVID-19 as well as for other endemic viruses and future virus outbreaks.

## Data availability statement

The original contributions presented in the study are included in the article/**Supplementary Material**, further inquiries can be directed to the corresponding author/s.

## Ethics statement

The studies involving humans were approved by the Dutch Central Committee on Research Involving Human Subjects (CCMO, NL7647.078.21), the Medical Research Ethics Committee from Erasmus University Medical Center (MEC-2021-0050) and the local review boards of all other participating centers. The studies were conducted in accordance with the local legislation and institutional requirements. The participants provided their written informed consent to participate in this study.

## Author contributions

LvL: Conceptualization, Formal analysis, Investigation, Methodology, Validation, Visualization, Writing – original draft, Writing – review & editing. MG: Conceptualization, Investigation, Methodology, Validation, Writing – original draft, Writing – review & editing. CG: Conceptualization, Investigation, Methodology, Writing – review & editing. PE: Conceptualization, Investigation, Methodology, Writing – review & editing. GdB: Conceptualization, Investigation, Methodology, Writing – review & editing. JP: Conceptualization, Investigation, Methodology, Writing – review & editing. AR: Conceptualization, Investigation, Methodology, Writing – review & editing. HJ: Conceptualization, Investigation, Methodology, Writing – review & editing. FV: Conceptualization, Investigation, Methodology, Writing – review & editing. MvG: Conceptualization, Formal analysis, Investigation, Methodology, Supervision, Writing – original draft, Writing – review & editing. RdV: Conceptualization, Formal analysis, Investigation, Methodology, Supervision, Writing – original draft, Writing – review & editing. VD: Conceptualization, Formal analysis, Investigation, Methodology, Supervision, Visualization, Writing – original draft, Writing – review & editing.

## Group members of VACOPID research group

Eric C.M. van Gorp^1,2^ MD PhD, Faye de Wilt^1^ BSc, Susanne Bogers^1^ MSc, Lennert Gommers^1^ BSc, Daryl Geers^1^ Marianne W. van der Ent^10^ MSc, P. Martin van Hagen^10,11^ MD PhD, Jelle W. van Haga^5^ BSc, Bregtje A. Lemkes^5^ MD PhD, Annelou van der Veen^5^ BSc, Rogier W. Sanders^3^ PhD, Karlijn van der Straten^3^ MD, Judith A. Burger^3^, BSc, Jacqueline van Rijswijk^3^ BSc, Khadija Tejjani^3^ BSc, Joey H. Bouhuijs^3^ BSc, Karina de Leeuw^7^ MD PhD, Annick A.J.M. van de Ven^7^ MD PhD, S.F.J. de Kruijf-Bazen^6^ MSc, Pieter van Paassen^6^ MD PhD, Lotte Wieten^6^ PhD, Petra H. Verbeek-Menken^8^ BSc, Annelies van Wengen^8^ MSc, Anke H.W. Bruns^4^ MD PhD, Helen L. Leavis^4^ MD PhD, Stefan Nierkens^12,13^ PhD.

^12^ Center for Translational Immunology, UMC Utrecht, The Netherlands

^13^ Princess Máxima Center for Pediatric Oncology, Utrecht, The Netherlands
