# Supplementary material for: Immunogenicity of COVID-19 booster vaccination in IEI patients and their one year clinical follow-up after start of the COVID-19 vaccination program
Source: Front Immunol. 2024 Apr 18;15:1390022. doi: 10.3389/fimmu.2024.1390022 (PMC11063285; doi:10.3389/fimmu.2024.1390022)
Supplement: Supplementary file 1 [file DataSheet_1.docx]

**supplementary material**

Supplement to:

**Immunogenicity of COVID-19 booster vaccination in IEI patients and their one year clinical follow-up after start of the COVID-19 vaccination program**

This appendix has been provided by the authors to give readers additional information about the work.

**Table of content:**

[Supplementary methods 3](#_Toc162623049)

[In- and exclusion criteria 3](#_Toc162623050)

[Study visits 4](#_Toc162623051)

[Evaluation of humoral response 6](#_Toc162623052)

[Evaluation of cellular response 7](#_Toc162623053)

[Supplementary table 1 8](#_Toc162623054)

[Supplementary figure 1 9](#_Toc162623055)

[Supplementary figure 2 10](#_Toc162623056)

[Supplementary figure 3 11](#_Toc162623057)

[Supplementary figure 4 12](#_Toc162623058)

[References 13](#_Toc162623059)

# **Supplementary methods**

## **In- and exclusion criteria**

This study is designed as a prospective, controlled multicenter cohort study aimed to assess the immunogenicity of the SARS-CoV-2 vaccine in patients with various primary immunodeficiencies (PIDs) compared to controls. To be eligible for participation, individuals must meet the following inclusion criteria:

- Eligible for COVID-19 vaccination according to the manufacturer's guidelines.
- Age of 18 years or older.
- Ability to comprehend the study's purpose and risks, providing fully informed and documented written consent.
- Diagnosis based on one of the International Union of Immunological Societies (IUIS) criteria, including Common Variable Immunodeficiency (CVID) with or without the use of immunosuppressive therapy, Combined Immunodeficiency (CID), Chronic Granulomatous Disease (CGD), X-linked Agammaglobulinemia (XLA), Selective IgG subclass deficiency, or Specific Antibody Deficiency with Normal Immunoglobulins (SADNI). Additionally, partners, siblings, or other family members of the patient (without a PID) are eligible.

Potential participants meeting any of the following criteria will be excluded from the study:

- History of severe adverse reactions linked to a vaccine or a severe allergic reaction (e.g., anaphylaxis) to any component of the study intervention(s).
- Pregnant or breastfeeding women.
- Active (hematological) malignancy.
- Human Immunodeficiency Virus (HIV) infection.
- Bleeding diathesis or a condition associated with prolonged bleeding that, in the investigator's opinion, would contraindicate intramuscular injection.

## **Study visits**

| Visit | 1 | 2 | 3 | 4 | 5A* | 5B* | 6 | End of study |
| --- | --- | --- | --- | --- | --- | --- | --- | --- |
| Description | Baseline measurements + vaccination 1 (March 2021) | Vaccination 2 | +28 days after vaccination 2 | +6 months after vaccination 2 | +/- 5 weeks after third vaccination | +/- 8 weeks after booster-vaccination | +/- 7 weeks second booster vaccination (including first booster vaccination in participants that received a third vaccination) | End of April 2020 |
| Informed consent | x |  |  |  | x (due to amendment) | x (due to amendment) | x (due to amendment) |  |
| Inclusion / exclusion criteria | x |  |  |  |  |  |  |  |
| Medical history, Concomitant medication, height/weight, vital signs and other baseline characteristics | x |  |  |  |  |  |  |  |
| SARS-COV-2 vaccination | mRNA-1273 COVID-19 | mRNA-1273 COVID-19 |  |  | Third vaccination (mRNA based) administered at public vaccination sites | mRNA-based booster vaccination administered at public vaccination sites | mRNA-based booster vaccination administered at public vaccination sites |  |
| Nasopharyngeal swab | x |  |  |  |  |  |  |  |
| Blood collection | x | x | x | x | x | x | x |  |
| Questionnaire focused on SARS-CoV-2 infections | x | x | x | x | x | x | x | x |

* Study visit 5 is divided into part A and B. Part A are participants receiving a third vaccination. In our study, these participants are CVID patients who use immunosuppressive drugs, or in specific individual cases when a medical specialist had reasonable arguments to make an exception to the aforementioned indications, based on proven or assumed non-response. Part B are participants who received a regular booster vaccination.

## **Evaluation of humoral response**

**Luminex assay**

A recombinant prefusion ectodomain trimer of the SARS-CoV-2 Spike (S) protein and the monomeric receptor binding domain (RBD) of the S protein were designed, manufactured, and purified as previously described (1). SARS-CoV-2 N protein (2) was kindly provided by Gestur Vidarsson and Federica Linty of Sanquin Research, Amsterdam, the Netherlands. The protein was covalently linked to Luminex Magplex beads, as previously outlined (3). For the assessment of IgG binding to the S protein, serum dilutions (100.000-fold) were mixed with the protein-coupled beads and incubated overnight on a shaker at 4°C. Subsequently, the plates were washed with TBS containing 0.05% Tween-20, followed by incubation with Goat-anti-human IgG-PE (SouthernBiotech) for 2 hours. Read-out was executed on a MAGPIX (Luminex) (3). The WHO International Standard for anti-SARS-CoV-2 immunoglobulin (NIBSC 20/136) was applied to convert the Median Fluorescence Intensity (MFI) output into binding antibody units per ml (BAU/ml). To validate assay performance, each plate included a titration of serum from a convalescent COVID-19 patient, along with positive and negative controls. In addition, 15 to 20% of samples of each run were duplicated to verify the results.

**Pseudovirus neutralization assay**

Neutralization activity was assessed using a pseudovirus neutralization assay, as previously described (4). Briefly, HEK293T/ACE2 cells were seeded at a density of 20,000 cells/well in a 96-well plate coated with 50 μg/mL poly-L-lysine one day before initiating the neutralization assay (5). Serum samples, heat inactivated and diluted 1:100, underwent 3-fold serial dilution in cell culture medium (DMEM (Gibco), supplemented with 10% FBS, penicillin (100 U/mL), streptomycin (100 μg/mL) and GlutaMax (Gibco)) and were incubated for 1 hour at 37°C with SARS-CoV-2 pseudovirus in a 1:1 ratio. SARS-CoV-2 pseudoviruses were produced by co-transfecting the pCR3 SARS-CoV-2-SΔ19 (Wuhan-Hu-1, GenBank MN908947.3 with amino acid substitution D614G) expression plasmid with the pHIV-1NL43 ΔEnv-NanoLuc reporter virus plasmid in HEK293T cells (ATCC, CRL-11268) in a 3:1 ratio and harvested 48 hours post transfection (5). Subsequently, the mixture of serum and pseudovirus were added to the cells in a 1:1 ratio and incubated for 48 hours at 37°C, followed by lysis buffer to measure the luciferase activity in cell lysates using the Nano-Glo Luciferase Assay System (Promega) and GloMax system (Turner BioSystems). Relative luminescence units (RLU) were normalized to the positive control wells where cells were infected with pseudovirus in the absence of sera.

Neutralization titers (ID50) were determined as the serum dilution at which infectivity was inhibited by 50%, respectively, using a non-linear regression curve fit (GraphPad Prism software version 8.3) and converted into international units per ml (IU/ml) using the WHO International Standard for anti-SARS-CoV-2 immunoglobulin (NIBSC 20/136). Samples with virus neutralization titers of < 2 IU/ml were defined as having undetectable neutralization.

## **Evaluation of cellular response**

**IFNg release assay (IGRA)**

The T cell response specific to SARS-CoV-2 was measured by using a commercially available IFNg Release Assay (IGRA, QuantiFERON, Qiagen) in whole blood following previously described methods and adhering to the manufacturer’s description (6). In short, heparinized whole blood was incubated with two different SARS-CoV-2 antigens for 20-24h, using a combination of peptides stimulating both CD4+ and CD8+ T-cells (Ag1, Ag2, QuantiFERON, QIAGEN). Following incubation, plasma was collected and IFNg production in response to the antigens was measured by ELISA. Results are presented in IU IFNg/ml after subtraction of the NIL control values as interpolated from a standard calibration curve. The lower limit of detection in this assay is set at 0.01 IU/ml, and the responder cut-off is set at 0.15 IU/ml.

# **Supplementary table 1**

| Supplementary table 1: Overview of non-infectious complications in CVID patients receiving a third dose of COVID-19 vaccine (third vaccination or booster) without a history of COVID-19 | |
| --- | --- |
| **No non-infectious complication (N=29)** | 29 (41.4%) |
| **Single non-infectious complication (N=18)** |  |
| Autoimmune cytopenia | 1 (1.4%) |
| Enteropathy | 4 (5.7%) |
| GLILD | 2 (2.9%) |
| Lymphoproliferative disease | 1 (1.4%) |
| Malignancy | 1 (1.4%) |
| Organ specific autoimmunity | 2 (2.9%) |
| Systemic autoimmunity | 2 (2.9%) |
| Other Granulomatous disease | 1 (1.4%) |
| Other | 4 (5.7%) |
| **Multiple non-infectious complications (N=23)** |  |
| Autoimmune cytopenia, Enteropathy, GLILD | 1 (1.4%) |
| Autoimmune cytopenia, Enteropathy, Other | 1 (1.4%) |
| Autoimmune cytopenia, GLILD, Other Granulomatous disease | 1 (1.4%) |
| Autoimmune cytopenia, Lymphoproliferative disease, GLILD | 2 (2.9%) |
| Autoimmune cytopenia, Lymphoproliferative disease, GLILD, Other | 1 (1.4%) |
| Autoimmune cytopenia, Malignancy, Lymphoproliferative disease | 2 (2.9%) |
| Autoimmune cytopenia, Organ specific autoimmunity, Enteropathy | 1 (1.4%) |
| Autoimmune cytopenia, Organ specific autoimmunity, GLILD | 1 (1.4%) |
| Autoimmune cytopenia, Systemic autoimmunity, Lymphoproliferative disease | 1 (1.4%) |
| Enteropathy, GLILD | 1 (1.4%) |
| Enteropathy, Lymphoproliferative disease | 1 (1.4%) |
| Lymphoproliferative disease, GLILD, Other Granulomatous disease | 1 (1.4%) |
| Lymphoproliferative disease, Other Granulomatous disease, Other | 1 (1.4%) |
| Malignancy, Lymphoproliferative disease | 1 (1.4%) |
| Organ specific autoimmunity, Lymphoproliferative disease | 2 (2.9%) |
| Organ specific autoimmunity, Lymphoproliferative disease, GLILD | 1 (1.4%) |
| Organ specific autoimmunity, Malignancy, GLILD | 1 (1.4%) |
| Organ specific autoimmunity, Malignancy, GLILD, Other | 1 (1.4%) |
| Organ specific autoimmunity, Malignancy, Lymphoproliferative disease | 1 (1.4%) |
| Systemic autoimmunity, Lymphoproliferative disease | 1 (1.4%) |

# **Supplementary figure 1**

**
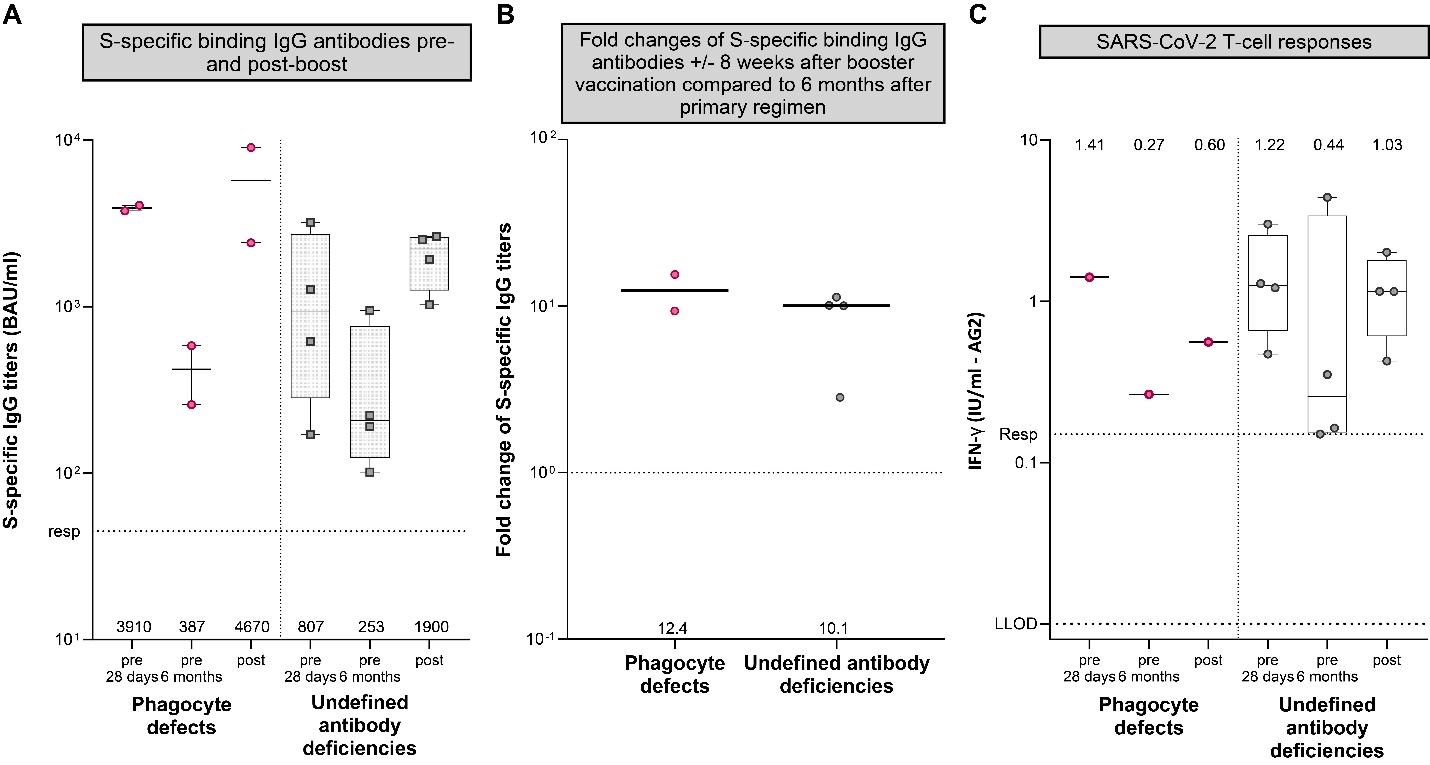
**

**Supplementary figure 1: S-specific IgG, fold changes, and SARS-CoV-2 specific T-cell responses in participants with phagocyte defects and undefined antibody deficiencies. (A)** S-specific IgG measured by 28 days after second vaccination, six months after the second vaccination, and eight weeks after booster vaccination. The number of participants per cohort correspond to table 1. Results are expressed in binding antibody units per milliliter (BAU/mL). The dotted line is the pre-defined responder cut-off (resp). Data are presented in box-and-whisker plots. The horizontal lines of the box-and-whisker plots indicate the median, the bounds of the boxes indicate the interquartile range, and the whiskers indicate the range. The numbers below the box-and-whisker plots indicate the geometric mean titers (GMT) per time point. Participants not using immunoglobulin replacement therapy (IGRT) are shown as circles, participants using IGRT are shown as squares. IgG titers were compared per cohort using the Wilcoxon paired signed rank test. **(B)** Fold changes of IgG antibodies post-boost (+/- 8 weeks after booster vaccination) compared to 6 months after primary regimen. The horizontal lines indicate the median, the whiskers indicate the interquartile range. All data points are shown. The dashed line represents a fold change of 1, where the titer at 6 months after primary regimen is equal to the titer after booster. All data points above the dashed line represents a fold increase, all data points below the dashed line a fold decrease. The numbers below indicate the median fold change. **(C)** SARS-CoV-2-specific T-cell responses measured by an interferon γ (IFN-γ) release assay (QIAGEN) after stimulation of whole blood 28 days and six months after second vaccination and eight weeks after booster vaccination. Lower limit of detection (LLOD) is .01 IU/ml and responder cut off (resp) is .15 IU/ml. Results are expressed as international units/milliliter (IU/mL). The dotted line is the pre-defined responder cut-off (resp). Data is presented in box-and-whisker plots. The horizontal lines of the box-and-whisker plots indicate the median, the bounds of the boxes indicate the interquartile range , and the whiskers indicate the range. All data points are shown. The numbers above the box-and-whisker plots indicate the geometric mean titer (GMT). Within each cohort, IFN-γ titers at 28 days and six months were compared using Wilcoxon paired signed rank test. The SPAD cohort is indicated with white symbols while the IgG cohort is indicated with orange symbols. Color coding is the same in all figures.

# **Supplementary figure 2**


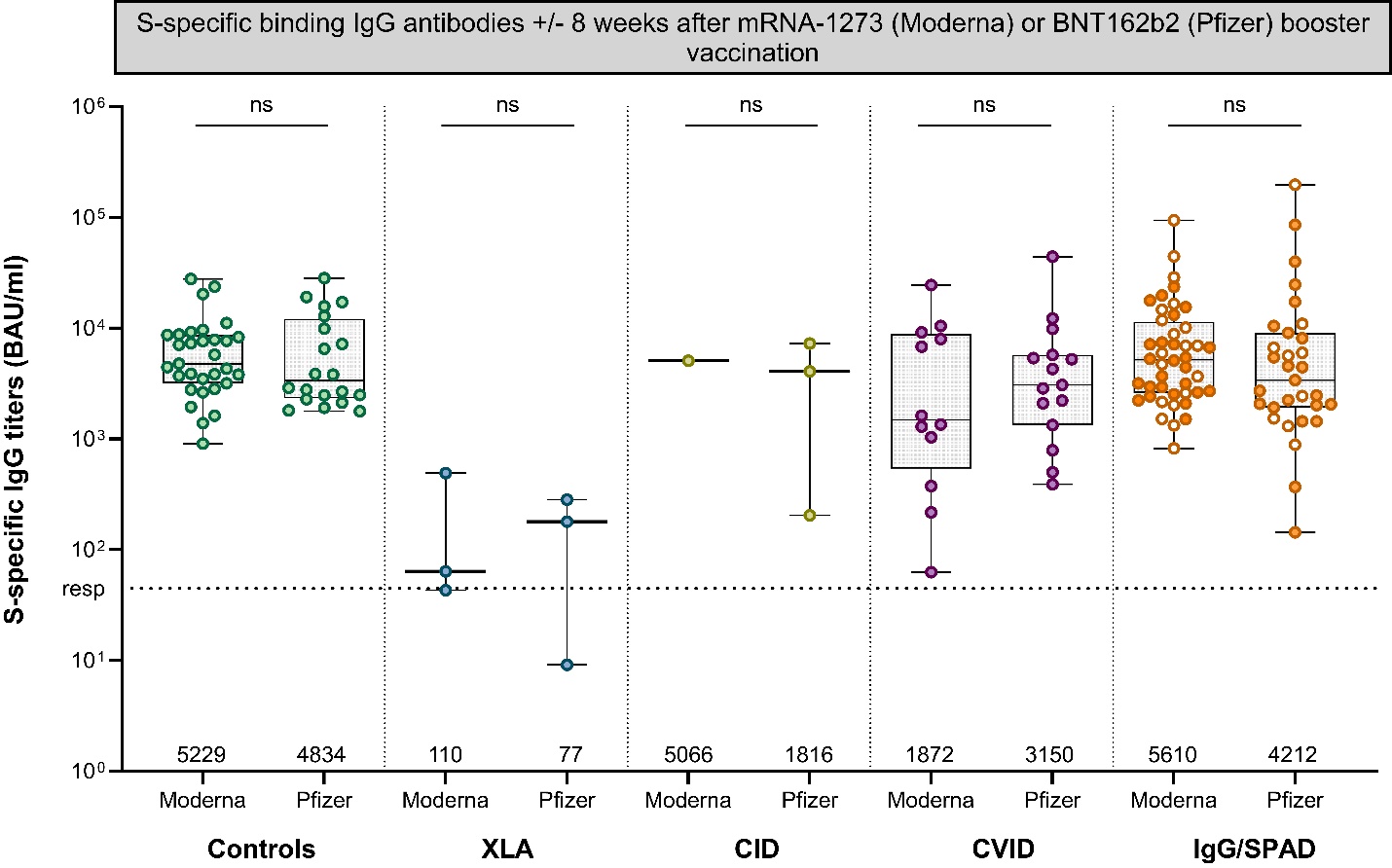


**Supplementary figure 2: S-specific IgG 8 weeks after booster vaccination stratified by booster type: mRNA-1273 (Moderna) or BNT162b2 (Pfizer).** S-specific IgG measured by Luminex for controls and different cohorts of inborn errors of immunity (IEI) patients eight weeks after booster vaccination. Results are expressed in binding antibody units per milliliter (BAU/mL). The dotted line is the pre-defined responder cut-off (resp). The horizontal lines of the box-and-whisker plots indicate the median, the bounds of the boxes indicate the interquartile range, and the whiskers indicate the range. All data points are shown. The numbers below the box-and-whisker plots indicate the geometric mean titers (GMT). IgG titers were compared per cohort using the Wilcoxon rank-sum test. The SPAD cohort is indicated with white symbols (with orange borders) while the IgG cohort is indicated with orange symbols. Color coding is the same in all figures. S = Spike, XLA = X-linked agammaglobulinemia, CID = Combined Immunodeficiency, CVID = Common Variable Immunodeficiency, IgG = Isolated IgG subclass deficiency ± IgA deficiency, SPAD = Specific polysaccharide antibody deficiency, ns = not significant.

# **Supplementary figure 3**


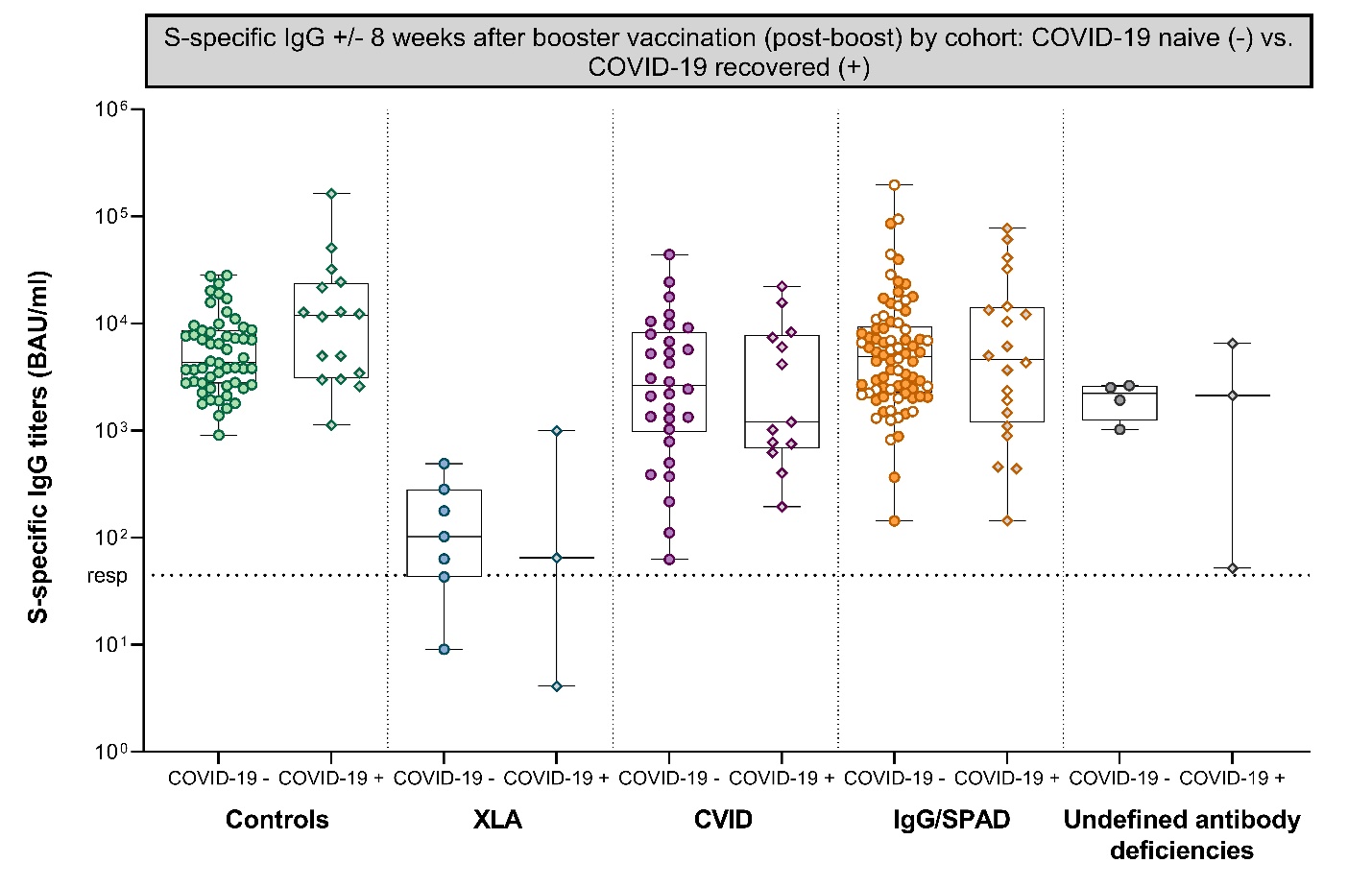


**Supplementary figure 3: S-specific IgG 8 weeks after booster vaccination by cohort stratified on COVID-19 naïve (-) or COVID-19 recovered (+)** S-specific IgG measured by custom Luminex assay for participants with (COVID-19 +, diamonds) and without (COVID-19 -, circles) a prior SARS-CoV-2 infection from the controls, XLA, CVID and IgG/SPAD cohort at +/- 8 weeks after booster vaccination. Results are expressed in binding antibody units per milliliter (BAU/mL). The dotted line is the pre-defined responder cut-off (resp). Data is presented in box-and-whisker plots. The horizontal lines of the box-and-whisker plots indicate the median, the bounds of the boxes indicate the interquartile range, and the whiskers indicate the range. All datapoints are shown. The numbers below the box-and-whisker plots indicate the geometric mean titer (GMT). IgG titers after booster vaccination between prior infected and not prior infected participants were compared using the Wilcoxon paired signed rank test. The SPAD cohort is indicated with white symbols while the IgG cohort is indicated with orange symbols. S = Spike, XLA = X-linked agammaglobulinemia, CVID = Common Variable Immunodeficiency, IgG = Isolated IgG subclass deficiency ± IgA deficiency, SPAD = Specific polysaccharide antibody deficiency.

# **Supplementary figure 4**


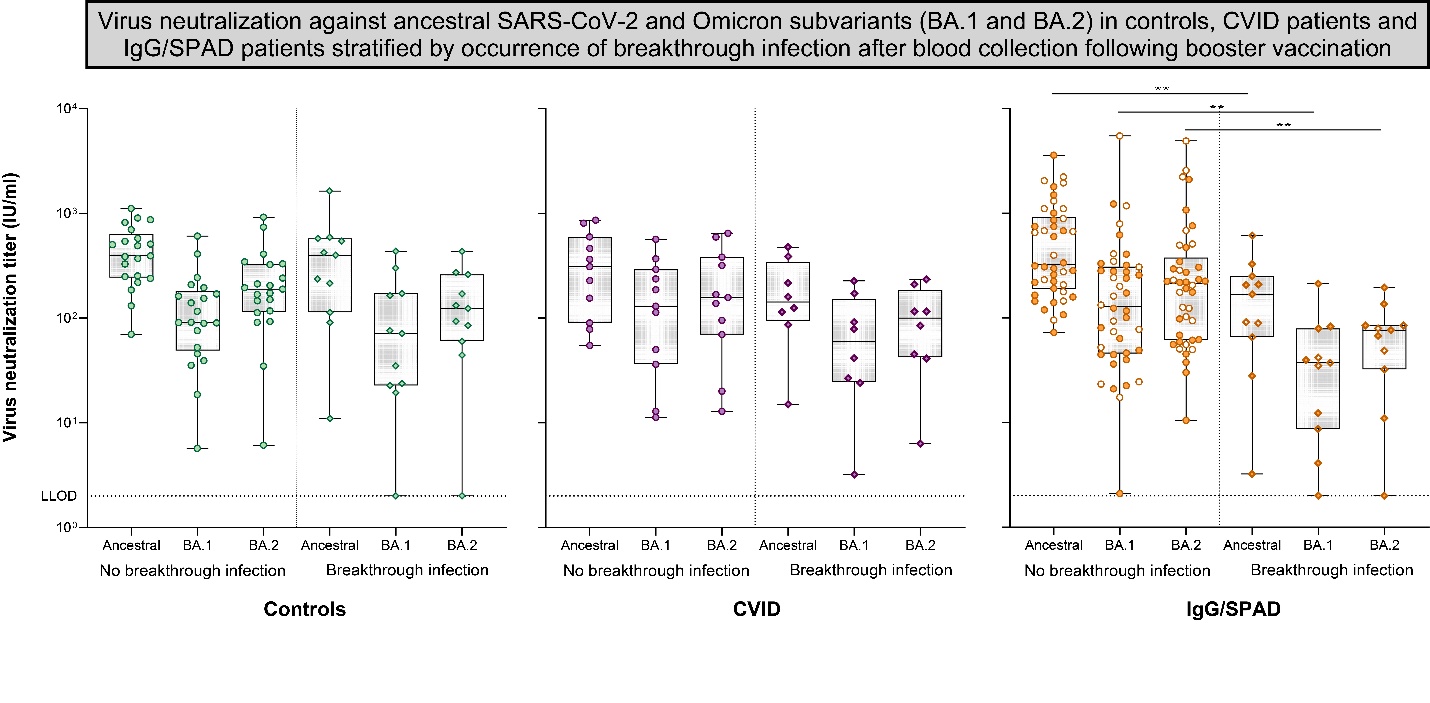
**Figure 4: SARS-CoV-2 neutralizing antibodies against ancestral SARS-CoV-2 and Omicron subvariants (BA.1 and BA.2) in participants without a history of SARS-CoV-2 infections.** Participants without a history of SARS-CoV-2 infection at the time of blood collection after booster vaccination were selected for determination of neutralizing antibodies. A proportion of these participants developed a breakthrough infection in the initial months after blood collection. Others reported no breakthrough infections after blood collection up to the end of the study. These two groups were compared using the Wilcoxon paired signed rank test. Mean follow-up time of the groups with and without breakthrough infection was similar. Neutralizing antibodies against ancestral SARS-CoV-2 and the by then dominant omicron variants BA.1 and BA.2 were determined. The virus neutralization titer (VNT) was given a value of 20 when no neutralization was observed (LLOD). Data is presented in box-and-whisker plots. The horizontal lines of the box-and-whisker plots indicate the median, the bounds of the boxes indicate the interquartile range, and the whiskers indicate the range. All datapoints are shown. The numbers below the box-and-whisker plots indicate the geometric mean titer (GMT). The SPAD cohort is indicated with white symbols while the IgG cohort is indicated with orange symbols. CVID = Common Variable Immunodeficiency, IgG = Isolated IgG subclass deficiency ± IgA deficiency, SPAD = Specific polysaccharide antibody deficiency, LLOD = lower limit of dection, ** = P<0.01.

# **References**

1. Grobben M, van der Straten K, Brouwer PJ, Brinkkemper M, Maisonnasse P, Dereuddre-Bosquet N, et al. Cross-reactive antibodies after SARS-CoV-2 infection and vaccination. Elife. 2021;10.

2. Larsen MD, de Graaf EL, Sonneveld ME, Plomp HR, Nouta J, Hoepel W, et al. Afucosylated IgG characterizes enveloped viral responses and correlates with COVID-19 severity. Science. 2021;371(6532).

3. Keuning MW, Grobben M, de Groen AC, Berman-de Jong EP, Bijlsma MW, Cohen S, et al. Saliva SARS-CoV-2 Antibody Prevalence in Children. Microbiol Spectr. 2021;9(2):e0073121.

4. Caniels TG, Bontjer I, van der Straten K, Poniman M, Burger JA, Appelman B, et al. Emerging SARS-CoV-2 variants of concern evade humoral immune responses from infection and vaccination. Sci Adv. 2021;7(36):eabj5365.

5. Schmidt F, Weisblum Y, Muecksch F, Hoffmann HH, Michailidis E, Lorenzi JCC, et al. Measuring SARS-CoV-2 neutralizing antibody activity using pseudotyped and chimeric viruses. J Exp Med. 2020;217(11).

6. Sanders JF, Bemelman FJ, Messchendorp AL, Baan CC, van Baarle D, van Binnendijk R, et al. The RECOVAC Immune-response Study: The Immunogenicity, Tolerability, and Safety of COVID-19 Vaccination in Patients With Chronic Kidney Disease, on Dialysis, or Living With a Kidney Transplant. Transplantation. 2021.
